# Allogeneic Hematopoietic Cell Transplantation for Older Adults with Acute Myeloid Leukemia

**DOI:** 10.3390/cancers10060179

**Published:** 2018-06-04

**Authors:** Jodi J. Lipof, Kah Poh Loh, Kristen O’Dwyer, Jane L. Liesveld

**Affiliations:** James P. Wilmot Cancer Institute, University of Rochester Medical Center, 601 Elmwood Avenue, P.O. Box 704, Rochester, NY 14642, USA; jodi_lipof@urmc.rochester.edu (J.J.L); Kahpoh_Loh@urmc.rochester.edu (K.P.L.); Kristen_odwyer@urmc.rochester.edu (K.O.)

**Keywords:** allogeneic stem cell transplantation, older adults, acute myeloid leukemia

## Abstract

Acute myeloid leukemia (AML) is a disease that affects adults aged 65 years and above, and survival in this population is poor. Allogeneic hematopoietic cell transplantation (allo-HCT) is a potentially curative therapy for these patients but is underutilized due to frequent comorbidities and perceived higher risk of treatment-related mortality and non-relapse mortality. Increasing data supports the utility of allo-HCT in fit older patients after intensive chemotherapy resulting in improvement of outcomes. With the development of reduced intensity and non-myeloablative conditioning regimens that are associated with lower rates of treatment-related toxicity and mortality, this has allowed more older patients with AML to receive allo-HCT. In this review, we provide some guidance on appropriate selection of older patients as transplant candidates, benefits and risks associated with allo-HCT, conditioning regimen choice, and stem cell transplant sources as they relate to the conduct of stem cell transplantation in older patients.

## 1. Introduction

**Figure 1:**
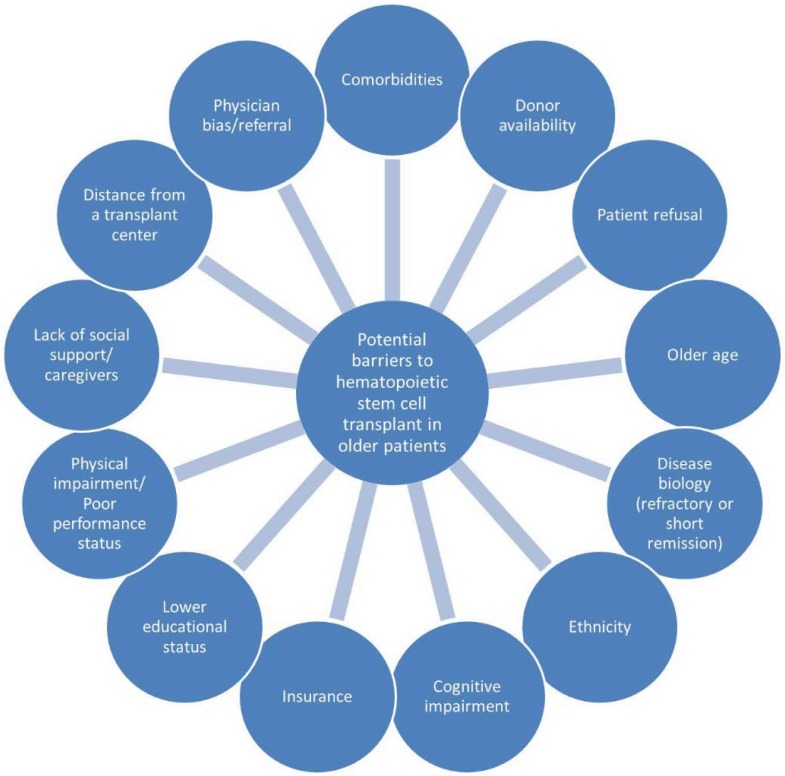
Potential barriers to hematopoietic stem cell transplantation in older patients.

Acute myeloid leukemia (AML) is a disease that primarily affects adults aged 65 years and above; the median age of patients at diagnosis is 68 years [1]. The estimated five-year survival in older adults with AML is poor at less than 5% [2]. The dismal prognosis in an older population is attributed to a variety of host and disease factors including high prevalence of comorbidity, frailty, poor functional status, unfavorable cytogenetics, greater resistance to leukemia-directed treatment, and higher rate of treatment-related toxicities [3,4]. Older patients are also more likely to have secondary AML related to antecedent hematologic disorders with consequent poor response to standard chemotherapy [5]. Allogeneic hematopoietic cell transplantation (allo-HCT) is a potentially curative therapy for patients with intermediate- or high-risk AML or those who fail to enter complete remission after one to two cycles of induction chemotherapy [6]. Older patients, historically, had not been considered for allo-HCT due to frequent comorbidities and perceived higher risk of treatment-related mortality and non-relapse mortality [7]. Recently, however, there is increasing data that support the utility of allo-HCT in fit older patients after intensive chemotherapy resulting in improvement of outcomes [8]. Despite this, less than 6% of older patients receive allo-HCT, which is partially attributed to the reluctance of physicians to transplant these patients and the challenges associated with the selection of transplant candidates [9]. Figure 1 shows the possible barriers to hematopoietic stem cell transplantation in older patients with AML.

Myeloablative conditioning regimens are frequently used in allo-HCT, but they are sometimes associated with an unacceptable rate of treatment-related mortality in older adults. The development of reduced intensity and non-myeloablative conditioning regimens that are associated with lower rates of treatment-related toxicity and mortality, in place of myeloablative conditioning regimens, has allowed a higher number of older patients with AML to receive allo-HCT [10]. In addition to this, the availability of various donor sources increases the access and eligibility for hematopoietic stem cell transplantation. In the past, the utilization of allo-HCT relied heavily and almost exclusively on stem cells harvested from matched sibling donors. However, the likelihood of finding a matched sibling donor is lower in the older AML population, as most of their sibling donors are older themselves and may have comorbidities that prohibit them from being donors. The alternative is usually a matched unrelated donor, which may pose difficulties for racial/ethnic minorities due to lower rates of locating acceptable donors in worldwide registries [11]. Because of this, other donor sources such as mismatched unrelated donors, haploidentical donors, and umbilical cord blood, are becoming increasingly available and utilized. In this review, we describe published studies that may guide the appropriate selection of older patients as transplant candidates. We also discuss the benefits and risks associated with allo-HCT, conditioning regimen choice, and stem cell transplant sources as they relate to conduct of stem cell transplantation in older patients. 

## 2. Selection of Candidates for Hematopoietic Stem Cell Transplantation 

According to the Center for International Blood Marrow Transplant Research (CIBMTR) database, the number of patients with AML aged 70 years and over who received allo-HCT increased from 0.1% in 2000 to 3.9% in 2013 [10]. This parallels the number of institutions that perform allo-HCT in older patients, which increased from 65 in 2000 to 93 in 2013 [10]. Some studies suggest improvements in outcomes of older patients with AML who received allo-HCT [10,12]. In the aforementioned CIBMTR database study, two-year progression-free and overall survival after allo-HCT improved from 22% and 26% during 2000–2007 to 32% and 39% during 2008–2013, respectively. There was no change in treatment-related mortality between the two time periods. In a European Group for Blood and Marrow Transplantation (EBMT) registry data study that included 1,333 patients aged 50 years and over with secondary AML and myelodysplastic syndrome (MDS) who received allo-HCT, there was no significant difference in overall survival and non-relapse mortality by age groups [13]. Four-year overall survival was 34% and 27%, respectively, in the patients aged 50 to 60 years and those over 60 years (*p* = 0.23). Four-year non-relapse mortality rate was 36% and 39% (*p* = 0.39), respectively [13]. These studies therefore support the feasibility, safety, and potential efficacy of allo-HCT in older patients. 

Historically, older patients with AML were excluded from consideration of hematopoietic stem cell transplantation solely based on chronological age. However, age alone is not a reliable predictor of a patient’s ability to tolerate intensive chemotherapy or allo-HCT. Instead, other factors such as comorbidities, functional status, and psychosocial support, in addition to disease characteristics need to be taken into account when considering treatment tolerance and outcomes [14,15,16]. For example, Sorror and colleagues developed the hematopoietic cell transplantation comorbidity index (HCT-CI) that may help risk stratify older patients with AML based on a number of medical comorbidities such as cardiac disease, cerebrovascular disease, diabetes, inflammatory bowel disease, pulmonary disease, psychiatric illness, obesity, infection, and other domains [17]. Patients are categorized into three risk groups: low (a score of 0), moderate (a score of 1 to 2), and high (a score of 3 or more). The two-year non-relapse mortality in the low, moderate, and high-risk groups was 14%, 21%, and 41%, respectively. The corresponding two-year overall survival was 71%, 60%, and 34%, respectively [17]. This tool therefore helps guide transplant evaluation, especially in older patients with a prevalence of these comorbidities [17]. There are also efforts to combine the HCT-CI with cytogenetic and molecular profiles or with clinical assessments to create an AML composite model. For example, Muffly and colleagues incorporated the HCT-CI as part of a geriatric assessment that included measures of frailty, functional status, and markers of inflammation/nutritional status [18]. The authors showed that the combination of geriatric assessment measures (specifically instrumental activity of daily living, gait speed, mental health, and C-reactive protein) and HCT-CI predicted poor outcomes in patients aged 60 years and over that underwent allo-HCT [18]. A geriatric assessment may also predict treatment toxicities and guide supportive care interventions, although further studies are needed in AML, especially as related to fitness for stem cell transplantation [19,20,21].

To improve the selection of candidates for allo-HCT, it is important to assess the physiological age and not rely on chronological age alone. The use of available tools such as the HCT-CI and geriatric assessment can help inform the risk of treatment-related mortality and overall survival [22]. A multi-disciplinary approach is needed when evaluating for transplant candidacy in this patient population with complex needs [23,24].

## 3. Conditioning Regimens

The choice of a conditioning regimen is an integral part of allo-HCT with influence on disease control and transplant-related toxicities. The CIBMTR classifies the conditioning regimens into myeloablative, reduced-intensity, and non-myeloablative categories [25,26]. Generally, myeloablative regimens induce irreversible pancytopenia and patients require support with hematopoietic stem cells. On the other hand, non-myeloablative regimens induce minimal cytopenias and patients do not require hematopoietic stem cell support. Reduced-intensity regimens fall in between myeloablative and non-myeloablative regimens, resulting in variable degrees of marrow ablation [25,26]. 

In older patients with AML, treatment with a myeloablative regimen for allo-HCT is associated with higher treatment-related mortality rates compared to those who did not undergo transplant [27]. Table 1 lists the studies that compared the various conditioning regimens and included some patients aged 60 and over [28,29,30,31,32,33]. It is worth noting that the upper age limit of patients included is usually 70 or lower. In the Dutch-Belgian Hemato-Oncology Cooperative Group and the Swiss Group for Clinical Cancer Research (HOVON-SAKK) collaborative study of 1032 patients, treatment-related mortality was higher in patients who underwent allo-HCT using a myeloablative conditioning regimen compared to those who did not undergo transplant (25% versus 4%, *p* < 0.01) [27]. This risk was higher in the older age group [Hazard Ratios (HR) 6.1, 95% Confidence Interval (CI) 3.0–12.2], compared to the younger age group (HR 2.7, 95% CI 1.5–4.9). Of note, only patients up to the age 55 were included in the study. Due to the high treatment-related mortality, attention has been shifted toward using reduced intensity and non-myeloablative conditioning regimens. A number of studies demonstrated a decrease in treatment-related mortality and toxicities including mucositis, hemorrhagic cystitis, cytomegalovirus infections (CMV), time to engraftment, and need for transfusions [28,31,34]. 

One concern, however, is the higher relapse rate associated with reduced-intensity regimens compared to myeloablative conditioning regimens [30]. It was postulated that the higher rates of relapse could be secondary to the decreased cytotoxic anti-leukemia effects, allowing persistence of quiescent leukemia stem cells or more overt minimal residual disease [35]. Published studies have reported variable results, with some demonstrating comparable overall survivals in those who received myeloablative and reduced-intensity conditioning regimens [32,33,36], and some reporting higher overall survivals in those who received myeloablative conditioning regimens [31,37]. The Blood and Marrow Transplant Clinical Trials Network (BMT CTN) 0901 study is the largest phase III randomized clinical trial (RCT) comparing outcomes by conditioning intensity in patients with high-risk AML and MDS [31]. In this RCT, 272 patients (median age 54.8 years, range 21.9–66.0) with a HCT-CI score of 4 or less were randomized to receive myeloablative or reduced-intensity conditioning regimens [31]. The trial was closed to accrual early due to a large difference noted in the relapse-free survival favoring the myeloablative arm. In patients with AML, compared to the reduced-intensity arm, the myeloablative arm has superior relapse-free survival (65.2% versus 45.3%; *p* < 0.01) and overall survival (76.4% versus 63.4%; *p* = 0.04) at 18 months. Treatment-related mortality at 18 months was higher in the myeloablative group compared to the reduced-intensity group (15.8% versus 4.4%; *p* < 0.02). Although treatment-related mortality was lower in the reduced-intensity group, this difference seems to have been offset by an increase in relapse rate and therefore a lower overall survival rate. Whether these results would be similar in studies examining only older recipients has not been explored in depth. In the BMT CTN 0901 study, the HRs for overall survival for myeloablative conditioning versus reduced-intensity conditioning did not appear to be different by age subgroups (≥55 versus <55 years, interaction *p* = 0.42).

Another large multicenter, retrospective study from the EBMT registry included 878 patients with AML or MDS who received allo-HCT utilizing myeloablative, reduced-intensity, or non-myeloablative conditioning regimens from 1998 to 2004 [33]. The myeloablative group was further divided into conventional (cyclophosphamide with high-dose busulfan) or hyperintense myeloablative (additional cytotoxic agents such as high-dose melphalan, thiotepa, etopisode, or cytarabine to conventional myeloablative regimen) groups. The follow-up period was up to 7 years after transplantation. In patients aged 50 and over, non-relapse mortality in the hyperintense myeloablative, conventional myeloablative, reduced-intensity, and non-myeloablative groups were 51%, 41%, 24%, and 31% (*p*-value comparing both myeloablative regimens versus both reduced-intensity and non-myeloablative group was <0.01), respectively. Relapse rates were 28%, 22%, 29%, and 49% (*p* = 0.20), and overall survivals were 21%, 41%, 56%, and 27% (*p*-value comparing reduced-intensity with other groups was <0.01), respectively. On multivariate analyses, older age (>50 versus ≤50 years) was significantly associated with a higher non-relapse mortality (HR 2.5, 95% CI 1.7–3.5) and lower overall survival (HR 1.9, 95% CI 1.1–3.1), but not relapse. As opposed to the BMT CTN 0901 study, this large retrospective study suggests potential lower non-relapse mortality, comparable relapse rate, and higher overall survival with reduced-intensity compared to conventional myeloablative conditioning regimens in older patients. Therefore, whether reduced-intensity versus myeloablative or non-ablative regimens lead to better survival in older patients with AML remains uncertain, and it is unlikely a randomized trial in older patients will be accomplished to answer this given the concern about high mortality rates with ablative regimens. Finally, a meta-analysis of eight studies included 6,464 AML/MDS patients who received myeloablative and reduced-intensity regimens. Of note, the BMT CTN 0901 was not included in this meta-analysis [38]. There was significant heterogeneity in the characteristics of patients included; patients in the reduced-intensity group were older, had lower HCT-CI scores, and had higher-risk disease based on cytogenetics. This reflected a selection bias with sicker patients being more likely to receive reduced-intensity regimens and healthier patients being more likely to receive myeloablative regimens. Despite this, there was no significant difference between the two groups in overall survival [Odds Ratio (OR) 0.96, 95% CI 0.84–1.08)], event-free survival (OR 0.88, 95% CI 0.77–1.00), or non-relapse mortality (OR 0.99, 95% CI 0.87–1.13).

Taken together, the BMT CTN 0901 study suggests that myeloablative conditioning regimens should be the standard of care for patients who are considered fit enough to tolerate this degree of intensity. For those who are perceived to be unfit for myeloablative regimens, reduced-intensity or non-myeloablative regimens may be considered. Alternatively, clinical trials or systemic agents such as chemotherapy without a transplant are reasonable in an unfit population. This population typically has a higher burden of comorbidities and decreased functional and performance status. 

## 4. Donor Sources for Older Transplant Recipients

Matched-related sibling donors are the main donor source for allo-HCT. However, the sibling match probability is decreased in older adults (28% in 65–74 year olds versus 39% in 45–64 year olds) [39]. Recent advances in HLA-typing have increased the pool of donor sources to include matched-unrelated, umbilical cord blood, haploidentical donors, and mismatched-unrelated donors (Table 2). The use of alternative donor sources increases the recipient eligibility pool and may reduce the time from diagnosis to transplant. Based on the National Marrow Donor Program (NMDP) registry, the likelihood of finding a matched-unrelated donor is 75% in white patients of European descent, 46% in white patients of Middle Eastern or North African descent, 16 to 19% in African Americans, and 27 to 52% in Hispanics, Asians, Pacific Islanders, and Native Americans [40]. Similarly, the likelihood of finding sufficient cord blood units (5/6 or 6/6 HLA-matched) is higher in patients of White European descent.

Studies comparing patients who received allo-HCT using matched-related donors versus matched-unrelated donors demonstrated comparable outcomes, although compared to young adults, older patients may have worse outcomes [45,46]. In a single-institution retrospective analysis of 405 patients (median age 54.5 years, range 20–72, 13% had a 9/10 HLA-matched donor) with various hematologic malignancies, outcomes including non-relapse mortality, relapse, and overall survival were not different in the matched-related and matched-unrelated donor groups [45]. Nevertheless, older patients had worse overall survival on multivariable analysis (≥50 versus <50 years; HR 1.4, 95% CI 1.0–1.9), but not non-relapse mortality and relapse. In a retrospective analysis of 368 older patients with AML (median age 57 years, range 50–73) from transplant centers of the German Cooperative Transplant Study Group, event-free survival, relapse, and overall survival were also similar between matched-related and matched-unrelated donors [46]. In this study, age as a continuous variable was not associated with event-free or overall survivals on multivariable analyses. However, older age was associated with higher relapse rate (HR 1.0, 95% CI 1.0–1.1).

The effect of donor age on transplant outcomes is an area of investigation. Some studies suggest a possible survival advantage when younger sibling donors were used as compared to older donors [47], while others did not show any difference in outcomes. For example, in a single-center retrospective study that included 442 patients (median 48, range 7–68), compared to patients who utilized a matched-unrelated donor, relapse rate was higher in those who utilized a matched-related donor aged 60 years and over (HR 4.41, 95% CI 1.52–12.80). This difference was not noted in patients who utilized a matched sibling donor aged less than 60 years of age (HR 1.18, 95% CI 0.55–1.55) [48]. The difference in outcomes may be due to the decreased allo-reactivity of older donor T cells that lead to a decrease in anti-leukemia effects [49]. In a second retrospective study using the EBMT registry that included 714 adults with AML (median age at transplant 61.4 years, range 55–74), outcomes such as relapse, non-relapse mortality, leukemia-free survival, and overall survival were similar in patients who received allo-HSCT from a matched-unrelated donor or a matched-related donor [50]. Taken together, matched-related donors and matched-unrelated donors are still the preferred donor sources, and the choice for one versus another depends on other factors related to the donor (age, smoking status, and comorbidity) and to patient-donor compatibility (7/8 or 8/8 HLA-matched, gender, ABO compatibility, and CMV status) [51].

A haploidentical transplantation is a technique that was initially limited by high transplant-related morbidity and mortality and engraftment failures [52,53]. With improvement in the depletion of T cells from the graft and unmanipulated in vivo regulation of T cells through post-infusion cyclophosphamide, haploidentical transplant is increasingly being utilized [43,54]. It is a very attractive option given most patients will have a relative (parents, siblings, or children) with one HLA-matching haplotype. In a retrospective study of 36 patients aged 60 and over with AML, three-year progression-free and overall survivals were 31% and 38%, respectively. In addition, older age was not associated with non-relapse morality, progression-free survival, and overall survival (60 to 69 and 70 to 75 versus 50 to 59). These were comparable to the study by the German Cooperative Transplant Study Group which utilized matched-related and matched-unrelated donors [46]. Currently, there is no RCT that compares the use of a haploidentical transplantation versus matched-related or unrelated transplant in older patients. A recently published retrospective study in 127 older patients with AML and MDS compared haploidentical transplantation with allo-HCT using matched-related or matched-unrelated donors. In the match-related, match-unrelated, and haploidentical groups, there were no significant differences in the two-year non-relapse mortality (17%, 23%, and 9%), relapse (32%, 34%, and 33%), and overall survival (62%, 55%, and 67%) [55]. Other studies in AML and MDS not limited to older patients also showed similar findings [42,56,57,58]. Therefore, existing studies support the use of haploidentical transplantation as an acceptable alternative to matched-related or unrelated transplant, especially in those who do not have readily available matched donors.

Umbilical cord blood transplantation is another reasonable alternative to matched-related and unrelated donor transplantation in the absence of available donors. Some of the benefits of cord blood transplantation include rapid availability and greater tolerance of HLA disparity [59,60]. However, as compared to haploidentical transplantation, cord blood transplantation is less utilized due to high rates of graft failure and delayed immune reconstitution. Nevertheless, it is feasible in older patients with AML, as shown in multiple studies [44,61,62]. In the CIBMTR Eurocord retrospective analysis that included 441 patients that received allo-HCT utilizing matched-related donors and 205 patients utilizing cord blood (over age 50), the transplant-related mortality was higher in the cord blood group than the 8/8 matched-unrelated donor group (35% versus 27%, *p* = 0.05), but no different than the 7/8 matched-unrelated donor group (35% versus 41%, *p* = 0.30) [61]. Three-year overall survival was lower in the cord blood group compared to the 8/8 matched-unrelated donor group (30% versus 43%, *p* < 0.01), and comparable to the 7/8 matched-unrelated donor group (30% versus 37%, *p* = 0.25). 

## 5. Graft-Versus-Host Disease in the Older Transplant Patient

Graft-versus-host disease (GVHD) is the most common transplant-related complication that affects patients who have undergone hematopoietic stem cell transplantation. GvHD occurs when the donor T-cells attack recipient tissues, affecting organs such as the skin, mouth, eyes, joints, gastrointestinal tract, liver, and lung. Acute GVHD generally occurs shortly after transplantation (within 100 days), whereas chronic GVHD typically occurs beyond 100 days of transplantation [63]. However, there are overlap features where acute GvHD may occur after 100 days and vice versa. A number of studies have shown that the risk of GVHD is increased with age [41,64,65]. GVHD is associated with decreased relapse risk due to graft-versus-leukemia effects, but not survival due to its associated treatment-related morbidity and mortality. Compared to their younger counterparts, older patients are especially susceptible to the consequences of GVHD due to their baseline lower physiologic reserve. In addition, GVHD can significantly affect quality of life, which is an important consideration in older patients [66,67,68,69]. Therefore, GVHD is an important factor when considering the conditioning regimens and donor sources as they are associated with variable risks of GVHD, which correlates with poor quality of life. One study suggests that older patients experience worse physical well-being but better social well-being compared to their younger counterparts [70]. Other studies are mixed suggesting that older patients may achieve better, comparable, or worse QoL than younger patients [71,72,73].

Compared to reduced-intensity or non-myeloablative regimens, myeloablative regimens have a higher incidence of acute and chronic GVHD, which is an important factor contributing to its high treatment-related morbidity and mortality rates [74,75]. In terms of donor sources, HLA-mismatched unrelated donors have the highest incidence of GVHD. Haploidentical transplantation has comparable or lower GVHD rates than matched-related donor transplantation [42,76,77,78]. Cord blood transplantation has a lower incidence of GVHD compared to match-related and unrelated donors [79,80]. Graft source (bone marrow versus peripheral-blood stem cells) also contributes to differing rates of GVHD, with a higher rate of chronic GVHD noted with peripheral-blood stem cell grafts [81,82]. However, engraftment failure is more common when a bone marrow graft is utilized, and relapse rates appear comparable.

High-dose steroids are typically the first-line of treatment for both acute and chronic GVHD. Steroids are associated with a myriad of side-effects such as hypertension, hyperglycemia, confusion/delirium, infections, bone loss, and easy bruising that can be problematic in older adults. Therefore, there is a need to balance the efficacy of steroids for controlling GVHD and the risks of steroids in this population. Steroid sparing agents can be utilized, but these may also be poorly tolerated in an older population due to immune suppression effects. Newer oral agents with activity in GVHD may be better tolerated in older patients (e.g., Janus kinase/JAK or Bruton’s tyrosine kinase/BTK inhibitors), but these have not been systematically studied in older patients [83,84,85]. 

## 6. Post-Transplant Relapse 

There are several strategies used in the post-transplant setting to prevent relapse of AML. This is especially important in older patients with AML given high rates of relapse. The first approach is typically reducing the doses of immunosuppressive agents to enhance graft versus leukemia effect. However, this can be difficult in patients with ongoing GVHD. Donor lymphocyte infusion is another strategy that can be given when there is increasing mixed-chimerism or in the setting of relapsed AML. DLI may be combined with lymphodepleting agents or systemic chemotherapy such as hypomethylating agents [86,87]. Once relapse has occurred after allo-grafting, survival is extremely poor (23% at one year) in all patients, with lower survival in older patients [88]. Few studies have specifically addressed relapse and responses to post-relapse treatments in older populations. Table 3 lists some of the therapies which have been utilized in cases of relapse in older transplant recipients. Because of the difficulty in treatment of relapse post-transplant, there is interest in use of maintenance therapies such as hypomethylating agents or targeted inhibitors such as FLT3 inhibitors in appropriate populations at high risk of relapse. The impact these interventions will have on older transplant recipients and their tolerability remain to be explored. Use of reduced intensity conditioning regimens in this population could increase risk of relapse, and many older patients may enter transplant with higher disease burden after hypomethylating agents or fewer consolidation cycles, but this has not been systematically studied. Table 3 lists representative regimens which might be used post-transplant to maintain or treat AML relapse. Most of these have not been systematically studied in an older patient population. There is evidence that detecting minimal residual disease early after transplant can be used to trigger strategies to prevent emerging relapse [89]. For example, in the RELAZA2 study, patients who had minimal residual disease detected after transplant were started on azacitidine [90]. In the 26% of patients who were minimal residual disease positive, 40% had a decline of MRD and 19% had stable MRD after azacitidine at 6 months. Relapse eventually occurred in 33% but the median day of relapse was delayed until 397 days.

Many of these regimens have not been specifically examined in an older population, but they represent a spectrum of what has been used for prevention or treatment of relapse in older individuals. Second transplant or intensive chemotherapy is rarely used in older adults to treat post-transplant relapse. Immunomodulatory drugs have been examined but are thought to be associated with increased risk of graft-versus-host disease in patients with acute myeloid leukemia.

## 7. Research Gaps and Ongoing Studies

There are many gaps in our knowledge in the field of allo-HCT for older patients with AML. To better guide the selection of older patients for allo-HCT, prospective trials need to incorporate patient-factors (e.g., physical function, comorbidity, nutritional status) and disease-factors (cytogenetic, molecular profile, etc.). There are also limited phase 3 randomized controlled trialss that compare the various conditioning regimens and alternative donor sources in older patients with AML. Multiple studies are ongoing that investigate various post-transplant maintenance strategies such as midostaurin (NCT02723435) or crenolanib (NCT01522469) in older patients with FLT3 activating mutations (NCT02723435). Although not limited to older patients, the BMT CTN 1506 study (NCT02997202) is currently investigating the use of gilteritinib as a maintenance therapy in patients with FLT3-internal tandem duplication AML. How best to treat GVHD in older transplant recipients has not been systemically studied and these studies have importance as GVHD may have greater impact on physical function in older transplant recipients [105]. Research on why elder AML patients are not referred for transplant evaluation is also needed as regards patient preference, hematologist/oncologist bias, access to transplant centers, and status of disease. Lower intensity treatments such as hypomethylating agents are often considered palliative, but they may be used as a bridge to allo-HCT for older patients given the relative low toxicity compared to intensive induction chemotherapy [106]. This strategy should be studied further.

## 8. Conclusions

Selected older patients with AML can benefit from allo-HCT, which provides the best chance for durable remission. Advances in the techniques and procedures of hematopoietic stem cell transplantation including alternate donor sources, reduced intensity conditioning regimens, and advanced strategies for preventing post-transplant relapse, have increased the availability and appropriateness of transplant for older adults with AML. Despite these advances, the number of older patients with AML who receive allo-HCT remains low. Further research is needed to improve candidate selection and access to donors, to optimize existing conditioning regimens, and to develop effective post-transplant maintenance strategies and treatment of relapsed disease.

## Figures and Tables

**Table 1:** Examples of studies that compared the various conditioning regimens and included some patients aged 60 and over.

Study	Study Design	*N*	Conditioning Regimens	Age, Median (Range)	OS	NRM/TRM, %	Relapse, %	Acute GVHD, %	Chronic GVHD, %
Aoudjhane 2005 [28]	Retrospective	722	MAC RIC	54 (50–64) 57 (50–73)	46 47 (2-year)	32 18 (2-year)	24 41 (2-year)	31 22 (Grade II-IV, 100-day)	56 48 (Any, 2-year)
Luger 2014 ^a^ [29]	Retrospective	5179 (AML: 3834)	MAC RIC BM RIC PBSC NMA	42 (18–68) 51 (19–69) 56 (18–70) 57 (18–70)	34 33 33 26 (5-year)	18 16 12 13 (3-month; 35% for all by year 5)	32 42 39 43 (5-year)	47 41 45 47 (Grade II-IV, 100-day)	41 31 45 41 (Any, 5-year)
Shimoni 2012 ^a^ [30]	Prospective	85 (AML: 50)	MAC RIC	49 (18–66) 60 (29–75)	50 44 (5-year)	18 20 (5-year)	40 47 (5-year)	37 25 (Grade II-IV, 5-year)	60 47 (Any, 5-year)
Scott 2017 ^a^ [31]	Randomized controlled trial	272 (AML: 218)	MAC RIC	55 (22–66) 55 (22–66)	76 ^b^ 63 ^b^ (18-month)	16 ^b^ 4 ^b^ (18-month)	16 ^b^ 51 ^b^ (18-month)	45 ^c^ 29 ^c^ (Grade II-IV, 100-day)	64 ^c^ 48 ^c^ (Any, 18-month)
Sebert 2015 [32]	Prospective	132	MAC RIC	44 (35–56) 54 (37–66)	43 50 (4-year)	28 21 (4-year)	33 38 (4-year)	61 35 (Grade II-IV, 120-day)	- -
Martino 2013 ^a^ [33]	Retrospective	878 (AML: 864)	HyperMAC ConvMAC RIC NMA	37 (18–60) 39 (21–63) 54 (19–70) 56 (41–72)	51 ^d^ 56 ^d^ 53 ^d^ 29 ^d^ (7-year)	27 ^e^ 22 ^e^ 18 ^e^ 29 ^e^ (7-year)	22 ^f^ 24 ^f^ 34 ^f^ 46 ^f^ (7-year)	29 27 25 30 (Any, 120-day)	47 44 48 50 (Any, 7-year)

Abbreviations: AML, acute myeloid leukemia; GVHD, graft-versus-host disease; MAC, myeloablative conditioning; NMA, non-myeloablative; NRM, non-relapse mortality; OS, overall survival; RIC, reduced-intensity conditioning; TRM, treatment-related mortality; BM, bone marrow; PBSC, peripheral blood stem cell; hyperMAC, hyper-intensive myeloablative conditioning; convMAC, conventional myeloablative conditioning. ^a^ Study included patients with acute myeloid leukemia and myelodysplastic syndrome. ^b^ Patients with acute myeloid leukemia. ^c^ Patients with acute myeloid leukemia and myelodysplastic syndrome. ^d^ For patients over the age of 50, 7-year overall survival in the hyperintense myeloablative, conventional myeloablative, reduced-intensity, and non-myeloablative groups were 21%, 41%, 27%, and 31%, respectively. ^e^ For patients over the age of 50, 7-year non-relapse mortality in the hyperintense myeloablative, conventional myeloablative, reduced-intensity, and non-myeloablative groups were 51%, 41%, 24%, and 31%, respectively. ^f^ For patients over the age of 50, 7-year relapse rates in the hyperintense myeloablative, conventional myeloablative, reduced-intensity, and non-myeloablative groups were 28%, 22%, 29%, and 49%, respectively.

**Table 2:** Benefits and limitations of the various donor sources.

Donor Source	Benefits	Limitations
Matched-sibling	Low rates of graft rejection. Lower risk of GVHD compared to unrelated donor	Low availability of donors, especially in the older population [39]
Matched-unrelated	Increased availability of donors.	Donor search time can be prolonged [40] May be extremely difficult to identify donor in ethnic/racial minorities [11] Higher rates of GVHD when compared to matched sibling [41]
Haploidentical	Increased and rapid availability of donors. Lower cost than UCB and unrelated donors Availability of donor for future lymphocyte/stem cell donations. Comparable overall survival, relapse rates, and non-relapse mortality to other donor sources [42]	Increased risk of graft rejection Risk of severe GVHD without adequate post-transplant prophylaxis (this is somewhat mitigated by use of post-transplant cyclophosphamide) [43]
Umbilical cord blood	Lower rates of acute and chronic GVHD Donor search time is lower than matched unrelated donors [11]	Delayed engraftment Increased risk of graft failure Lower quantity of harvest, need for adequate cell dose. Higher rates of infections due to slower engraftment and immune reconstitution [44] No availability of future donor lymphocytes/stem cells

**Table 3:** Representative Modalities Utilized Post-Transplant to Prevent or Treat Relapse.

Intervention	Examples
Early tapering of immune suppression [91]	
Graded donor lymphocyte infusion (DLI) dosing [92]	
Hypomethylating agents	Azacitidine [93,94,95]
	Decitabine [96]
Hypomethylating agents and DLI [97,98]	
FMS-like tyrosine kinase (FLT) 3 inhibitors	Sorafenib [99]
	Midostaurin [100]
Possibly B-cell lymphoma 2 (Bcl-2) inhibitors	Venetoclax [101]
Possibly Isocitrate dehydrogenase (IDH) 2 inhibitors	Enasidenib [102]
Possibly checkpoint inhibitors or cytotoxic T-lymphocyte-associated protein (CTLA) 4 antagonists	Ipilimumab [103]
Regulatory T cells (Tregs) with azacitidine [104]	

## References

[1] (2018). Seer Cancer Statistics Factsheets: Acute Myeloid Leukemia.

[2] Menzin J., Lang K., Earle C.C., Kerney D., Mallick R. (2002). The outcomes and costs of acute myeloid leukemia among the elderly. Arch. Intern. Med..

[3] Leith C.P., Kopecky K.J., Chen I.M., Eijdems L., Slovak M.L., McConnell T.S., Head D.R., Weick J., Grever M.R., Appelbaum F.R. (1999). Frequency and clinical significance of the expression of the multidrug resistance proteins MDR1/P-glycoprotein, MRP1, and LRP in acute myeloid leukemia: A southwest oncology group study. Blood.

[4] Oran B., Weisdorf D.J. (2012). Survival for older patients with acute myeloid leukemia: A population-based study. Haematologica.

[5] Koh Y., Kim I., Bae J.Y., Song E.Y., Kim H.K., Yoon S.S., Lee D.S., Park S.S., Park M.H., Park S. (2010). Prognosis of secondary acute myeloid leukemia is affected by the type of the preceding hematologic disorders and the presence of trisomy 8. Jpn. J. Clin. Oncol..

[6] Peccatori J., Ciceri F. (2010). Allogeneic stem cell transplantation for acute myeloid leukemia. Haematologica.

[7] Podoltsev N.A., Stahl M., Zeidan A.M., Gore S.D. (2017). Selecting initial treatment of acute myeloid leukaemia in older adults. Blood Rev..

[8] Devine S.M., Owzar K., Blum W., Mulkey F., Stone R.M., Hsu J.W., Champlin R.E., Chen Y.-B., Vij R., Slack J. (2015). Phase ii study of allogeneic transplantation for older patients with acute myeloid leukemia in first complete remission using a reduced-intensity conditioning regimen: Results from cancer and leukemia Group B 100103 (alliance for clinical trials in oncology)/blood and marrow transplant clinical trial network 0502. J. Clin. Oncol..

[9] Ustun C., Lazarus H.M., Weisdorf D. (2013). To transplant or not: A dilemma for treatment of elderly AML patients in the twenty-first century. Bone Marrow Transplant..

[10] Muffly L., Pasquini M.C., Martens M., Brazauskas R., Zhu X., Adekola K., Aljurf M., Ballen K.K., Bajel A., Baron F. (2017). Increasing use of allogeneic hematopoietic cell transplantation in patients aged 70 years and older in the united states. Blood.

[11] Majhail N.S., Nayyar S., Santibanez M.E., Murphy E.A., Denzen E.M. (2012). Racial disparities in hematopoietic cell transplantation in the united states. Bone Marrow Transplant..

[12] Rashidi A., Ebadi M., Colditz G.A., DiPersio J.F. (2016). Outcomes of allogeneic stem cell transplantation in elderly patients with acute myeloid leukemia: A systematic review and meta-analysis. Biol. Blood Marrow Transplant..

[13] Lim Z., Brand R., Martino R., van Biezen A., Finke J., Bacigalupo A., Beelen D., Devergie A., Alessandrino E., Willemze R. (2010). Allogeneic hematopoietic stem-cell transplantation for patients 50 years or older with myelodysplastic syndromes or secondary acute myeloid leukemia. J. Clin. Oncol..

[14] Pohlen M., Groth C., Sauer T., Gorlich D., Mesters R., Schliemann C., Lenz G., Muller-Tidow C., Buchner T., Berdel W.E. (2016). Outcome of allogeneic stem cell transplantation for AML and myelodysplastic syndrome in elderly patients (60 years). Bone Marrow Transplant..

[15] Modi D., Deol A., Kim S., Ayash L., Alavi A., Ventimiglia M., Bhutani D., Ratanatharathorn V., Uberti J.P. (2017). Age does not adversely influence outcomes among patients older than 60 years who undergo allogeneic hematopoietic stem cell transplant for AML and myelodysplastic syndrome. Bone Marrow Transplant..

[16] Armand P., Kim H.T., Logan B.R., Wang Z., Alyea E.P., Kalaycio M.E., Maziarz R.T., Antin J.H., Soiffer R.J., Weisdorf D.J. (2014). Validation and refinement of the disease risk index for allogeneic stem cell transplantation. Blood.

[17] Sorror M.L., Maris M.B., Storb R., Baron F., Sandmaier B.M., Maloney D.G., Storer B. (2005). Hematopoietic cell transplantation (HCT)-specific comorbidity index: A new tool for risk assessment before allogeneic HCT. Blood.

[18] Muffly L.S., Kocherginsky M., Stock W., Chu Q., Bishop M.R., Godley L.A., Kline J., Liu H., Odenike O.M., Larson R.A. (2014). Geriatric assessment to predict survival in older allogeneic hematopoietic cell transplantation recipients. Haematologica.

[19] Clough-Gorr K.M., Stuck A.E., Thwin S.S., Silliman R.A. (2010). Older breast cancer survivors: Geriatric assessment domains are associated with poor tolerance of treatment adverse effects and predict mortality over 7 years of follow-up. J. Clin. Oncol..

[20] Mohile S.G., Velarde C., Hurria A., Magnuson A., Lowenstein L., Pandya C., O’Donovan A., Gorawara-Bhat R., Dale W. (2015). Geriatric assessment-guided care processes for older adults: A delphi consensus of geriatric oncology experts. J. Natl. Compr. Cancer Netw..

[21] Extermann M., Boler I., Reich R.R., Lyman G.H., Brown R.H., DeFelice J., Levine R.M., Lubiner E.T., Reyes P., Schreiber F.J. (2012). Predicting the risk of chemotherapy toxicity in older patients: The chemotherapy risk assessment scale for high-age patients (crash) score. Cancer.

[22] Artz A.S. (2016). Biologic vs. physiologic age in the transplant candidate. Hematology Am Soc Hematol Educ Program.

[23] Rosko A., Artz A. (2017). Aging: Treating the older patient. Biol. Blood Marrow Transplant..

[24] Muffly L.S., Boulukos M., Swanson K., Kocherginsky M., Cerro P.D., Schroeder L., Pape L., Extermann M., Van Besien K., Artz A.S. (2013). Pilot study of comprehensive geriatric assessment (CGA) in allogeneic transplant: CGA captures a high prevalence of vulnerabilities in older transplant recipients. Biol. Blood Marrow Transplant..

[25] Jethava Y.S., Sica S., Savani B., Socola F., Jagasia M., Mohty M., Nagler A., Bacigalupo A. (2017). Conditioning regimens for allogeneic hematopoietic stem cell transplants in acute myeloid leukemia. Bone Marrow Transplant..

[26] Bacigalupo A., Ballen K., Rizzo D., Giralt S., Lazarus H., Ho V., Apperley J., Slavin S., Pasquini M., Sandmaier B.M. (2009). Defining the intensity of conditioning regimens: Working definitions. Biol. Blood Marrow Transplant..

[27] Cornelissen J.J., van Putten W.L., Verdonck L.F., Theobald M., Jacky E., Daenen S.M., van Marwijk Kooy M., Wijermans P., Schouten H., Huijgens P.C. (2007). Results of a HOVON/SAKK donor versus no-donor analysis of myeloablative HLA-identical sibling stem cell transplantation in first remission acute myeloid leukemia in young and middle-aged adults: Benefits for whom?. Blood.

[28] Aoudjhane M., Labopin M., Gorin N.C., Shimoni A., Ruutu T., Kolb H.J., Frassoni F., Boiron J.M., Yin J.L., Finke J. (2005). Comparative outcome of reduced intensity and myeloablative conditioning regimen in hla identical sibling allogeneic haematopoietic stem cell transplantation for patients older than 50 years of age with acute myeloblastic leukaemia: A retrospective survey from the acute leukemia working party (ALWP) of the european group for blood and marrow transplantation (EBMT). Leukemia.

[29] Luger S.M., Ringden O., Zhang M.J., Perez W.S., Bishop M.R., Bornhauser M., Bredeson C.N., Cairo M.S., Copelan E.A., Gale R.P. (2012). Similar outcomes using myeloablative vs reduced-intensity allogeneic transplant preparative regimens for AML or MDS. Bone Marrow Transplant..

[30] Shimoni A., Shem-Tov N., Volchek Y., Danylesko I., Yerushalmi R., Nagler A. (2012). Allo-SCT for AML and MDS with treosulfan compared with BU-based regimens: Reduced toxicity vs. reduced intensity. Bone Marrow Transplant..

[31] Scott B.L., Pasquini M.C., Logan B.R., Wu J., Devine S.M., Porter D.L., Maziarz R.T., Warlick E.D., Fernandez H.F., Alyea E.P. (2017). Myeloablative versus reduced-intensity hematopoietic cell transplantation for acute myeloid leukemia and myelodysplastic syndromes. J. Clin. Oncol..

[32] Sebert M., Porcher R., Robin M., Ades L., Boissel N., Raffoux E., Xhaard A., Dhedin N., Larghero J., Himberlin C. (2015). Equivalent outcomes using reduced intensity or conventional myeloablative conditioning transplantation for patients aged 35 years and over with AML. Bone Marrow Transplant..

[33] Martino R., de Wreede L., Fiocco M., van Biezen A., von dem Borne P.A., Hamladji R.M., Volin L., Bornhauser M., Robin M., Rocha V. (2013). Comparison of conditioning regimens of various intensities for allogeneic hematopoietic SCT using HLA-identical sibling donors in AML and MDS with <10% BM blasts: A report from EBMT. Bone Marrow Transplant..

[34] Ringden O., Erkers T., Aschan J., Garming-Legert K., Le Blanc K., Hagglund H., Omazic B., Svenberg P., Dahllof G., Mattsson J. (2013). A prospective randomized toxicity study to compare reduced-intensity and myeloablative conditioning in patients with myeloid leukaemia undergoing allogeneic haematopoietic stem cell transplantation. J. Intern. Med..

[35] Goyal G., Gundabolu K., Vallabhajosyula S., Silberstein P.T., Bhatt V.R. (2016). Reduced-intensity conditioning allogeneic hematopoietic-cell transplantation for older patients with acute myeloid leukemia. Ther. Adv. Hematol..

[36] Bornhauser M., Kienast J., Trenschel R., Burchert A., Hegenbart U., Stadler M., Baurmann H., Schafer-Eckart K., Holler E., Kroger N. (2012). Reduced-intensity conditioning versus standard conditioning before allogeneic haemopoietic cell transplantation in patients with acute myeloid leukaemia in first complete remission: A prospective, open-label randomised phase 3 trial. Lancet Oncol..

[37] McClune B.L., Weisdorf D.J., Pedersen T.L., Tunes da Silva G., Tallman M.S., Sierra J., Dipersio J., Keating A., Gale R.P., George B. (2010). Effect of age on outcome of reduced-intensity hematopoietic cell transplantation for older patients with acute myeloid leukemia in first complete remission or with myelodysplastic syndrome. J. Clin. Oncol..

[38] Zeng W., Huang L., Meng F., Liu Z., Zhou J., Sun H. (2014). Reduced-intensity and myeloablative conditioning allogeneic hematopoietic stem cell transplantation in patients with acute myeloid leukemia and myelodysplastic syndrome: A meta-analysis and systematic review. Int. J. Clin. Exp. Med..

[39] Besse K., Maiers M., Confer D., Albrecht M. (2016). On modeling human leukocyte antigen-identical sibling match probability for allogeneic hematopoietic cell transplantation: Estimating the need for an unrelated donor source. Biol. Blood Marrow Transplant..

[40] Gragert L., Eapen M., Williams E., Freeman J., Spellman S., Baitty R., Hartzman R., Rizzo J.D., Horowitz M., Confer D. (2014). HLA match likelihoods for hematopoietic stem-cell grafts in the U.S. Registry. N. Engl. J. Med..

[41] Flowers M.E., Inamoto Y., Carpenter P.A., Lee S.J., Kiem H.P., Petersdorf E.W., Pereira S.E., Nash R.A., Mielcarek M., Fero M.L. (2011). Comparative analysis of risk factors for acute graft-versus-host disease and for chronic graft-versus-host disease according to national institutes of health consensus criteria. Blood.

[42] Di Stasi A., Milton D.R., Poon L.M., Hamdi A., Rondon G., Chen J., Pingali S.R., Konopleva M., Kongtim P., Alousi A. (2014). Similar transplantation outcomes for acute myeloid leukemia and myelodysplastic syndrome patients with haploidentical versus 10/10 human leukocyte antigen-matched unrelated and related donors. Biol. Blood Marrow Transplant..

[43] Lee C.J., Savani B.N., Mohty M., Labopin M., Ruggeri A., Schmid C., Baron F., Esteve J., Gorin N.C., Giebel S. (2017). Haploidentical hematopoietic cell transplantation for adult acute myeloid leukemia: A position statement from the acute leukemia working party of the European society for blood and marrow transplantation. Haematologica.

[44] Sandhu K.S., Brunstein C., DeFor T., Bejanyan N., Arora M., Warlick E., Weisdorf D., Ustun C. (2016). Umbilical cord blood transplantation outcomes in acute myelogenous leukemia/myelodysplastic syndrome patients aged ≥ 70 years. Biol. Blood Marrow Transplant..

[45] Mielcarek M., Storer B.E., Sandmaier B.M., Sorror M.L., Maloney D.G., Petersdorf E., Martin P.J., Storb R. (2007). Comparable outcomes after nonmyeloablative hematopoietic cell transplantation with unrelated and related donors. Biol. Blood Marrow Transplant..

[46] Schetelig J., Bornhauser M., Schmid C., Hertenstein B., Schwerdtfeger R., Martin H., Stelljes M., Hegenbart U., Schafer-Eckart K., Fussel M. (2008). Matched unrelated or matched sibling donors result in comparable survival after allogeneic stem-cell transplantation in elderly patients with acute myeloid leukemia: A report from the cooperative German transplant study group. J. Clin. Oncol..

[47] Ayuk F., Zabelina T., Wortmann F., Alchalby H., Wolschke C., Lellek H., Bacher U., Zander A., Kroger N. (2013). Donor choice according to age for allo-SCT for AML in complete remission. Bone Marrow Transplant..

[48] Servais S., Porcher R., Xhaard A., Robin M., Masson E., Larghero J., Ribaud P., Dhedin N., Abbes S., Sicre F. (2014). Pre-transplant prognostic factors of long-term survival after allogeneic peripheral blood stem cell transplantation with matched related/unrelated donors. Haematologica.

[49] Friedman J.S., Alpdogan O., van den Brink M.R., Liu C., Hurwitz D., Boyd A., Kupper T.S., Burakoff S.J. (2004). Increasing T-cell age reduces effector activity but preserves proliferative capacity in a murine allogeneic major histocompatibility complex-mismatched bone marrow transplant model. Biol. Blood Marrow Transplant..

[50] Peffault de Latour R., Labopin M., Cornelissen J., Vigouroux S., Craddock C., Blaise D., Huyn A., Vindelov L., Maertens J., Chevallier P. (2015). In patients older than 55 years with AML in first CR, should we search for a matched unrelated donor when an old sibling donor is available?. Bone Marrow Transplant..

[51] Howard C.A., Fernandez-Vina M.A., Appelbaum F.R., Confer D.L., Devine S.M., Horowitz M.M., Mendizabal A., Laport G.G., Pasquini M.C., Spellman S.R. (2015). Recommendations for donor human leukocyte antigen assessment and matching for allogeneic stem cell transplantation: Consensus opinion of the blood and marrow transplant clinical trials network (BMT CTN). Biol. Blood Marrow Transplant..

[52] Powles R.L., Morgenstern G.R., Kay H.E., McElwain T.J., Clink H.M., Dady P.J., Barrett A., Jameson B., Depledge M.H., Watson J.G. (1983). Mismatched family donors for bone-marrow transplantation as treatment for acute leukaemia. Lancet.

[53] Beatty P.G., Clift R.A., Mickelson E.M., Nisperos B.B., Flournoy N., Martin P.J., Sanders J.E., Stewart P., Buckner C.D., Storb R. (1985). Marrow transplantation from related donors other than HLA-identical siblings. N. Engl. J. Med..

[54] Montoro J., Sanz J., Sanz G.F., Sanz M.A. (2016). Advances in haploidentical stem cell transplantation for hematologic malignancies. Leuk. Lymphoma.

[55] Bashey Z.A., Zhang X., Brown S., Jackson K., Morris L.E., Holland H.K., Bashey A., Solomon S.R., Solh M. (2018). Comparison of outcomes following transplantation with T-replete HLA-haploidentical donors using post-transplant cyclophosphamide to matched related and unrelated donors for patients with AML and MDS aged 60 years or older. Bone Marrow Transplant..

[56] Ciurea S.O., Zhang M.J., Bacigalupo A.A., Bashey A., Appelbaum F.R., Aljitawi O.S., Armand P., Antin J.H., Chen J., Devine S.M. (2015). Haploidentical transplant with posttransplant cyclophosphamide vs. matched unrelated donor transplant for acute myeloid leukemia. Blood.

[57] Rashidi A., DiPersio J.F., Westervelt P., Vij R., Schroeder M.A., Cashen A.F., Fehniger T.A., Romee R. (2016). Comparison of outcomes after peripheral blood haploidentical versus matched unrelated donor allogeneic hematopoietic cell transplantation in patients with acute myeloid leukemia: A retrospective single-center review. Biol. Blood Marrow Transplant..

[58] Rashidi A., Slade M., DiPersio J.F., Westervelt P., Vij R., Romee R. (2016). Post-transplant high-dose cyclophosphamide after HLA-matched vs. haploidentical hematopoietic cell transplantation for AML. Bone Marrow Transplant..

[59] Barker J.N., Krepski T.P., DeFor T.E., Davies S.M., Wagner J.E., Weisdorf D.J. (2002). Searching for unrelated donor hematopoietic stem cells: Availability and speed of umbilical cord blood versus bone marrow. Biol. Blood Marrow Transplant..

[60] Sanz J., Jaramillo F.J., Planelles D., Montesinos P., Lorenzo I., Moscardo F., Martin G., Lopez F., Martinez J., Jarque I. (2014). Impact on outcomes of human leukocyte antigen matching by allele-level typing in adults with acute myeloid leukemia undergoing umbilical cord blood transplantation. Biol. Blood Marrow Transplant..

[61] Weisdorf D., Eapen M., Ruggeri A., Zhang M.J., Zhong X., Brunstein C., Ustun C., Rocha V., Gluckman E. (2014). Alternative donor transplantation for older patients with acute myeloid leukemia in first complete remission: A center for international blood and marrow transplant research-eurocord analysis. Biol. Blood Marrow Transplant..

[62] Majhail N.S., Brunstein C.G., Shanley R., Sandhu K., McClune B., Oran B., Warlick E.D., Wagner J.E., Weisdorf D.J. (2012). Reduced-intensity hematopoietic cell transplantation in older patients with AML/MDS: Umbilical cord blood is a feasible option for patients without HLA-matched sibling donors. Bone Marrow Transplant..

[63] Nassereddine S., Rafei H., Elbahesh E., Tabbara I. (2017). Acute graft versus host disease: A comprehensive review. Anticancer Res..

[64] Jagasia M., Arora M., Flowers M.E., Chao N.J., McCarthy P.L., Cutler C.S., Urbano-Ispizua A., Pavletic S.Z., Haagenson M.D., Zhang M.J. (2012). Risk factors for acute GVHD and survival after hematopoietic cell transplantation. Blood.

[65] Hahn T., McCarthy P.L., Zhang M.J., Wang D., Arora M., Frangoul H., Gale R.P., Hale G.A., Horan J., Isola L. (2008). Risk factors for acute graft-versus-host disease after human leukocyte antigen-identical sibling transplants for adults with leukemia. J. Clin. Oncol..

[66] Pidala J., Kurland B., Chai X., Majhail N., Weisdorf D.J., Pavletic S., Cutler C., Jacobsohn D., Palmer J., Arai S. (2011). Patient-reported quality of life is associated with severity of chronic graft-versus-host disease as measured by NIH criteria: Report on baseline data from the chronic GVHD consortium. Blood.

[67] Sinding C., Wiernikowski J., Aronson J. (2005). Cancer care from the perspectives of older women. Oncol. Nurs. Forum.

[68] Lee S.J., Kim H.T., Ho V.T., Cutler C., Alyea E.P., Soiffer R.J., Antin J.H. (2006). Quality of life associated with acute and chronic graft-versus-host disease. Bone Marrow Transplant..

[69] Silvestri G., Pritchard R., Welch H.G. (1998). Preferences for chemotherapy in patients with advanced non-small cell lung cancer: Descriptive study based on scripted interviews. BMJ.

[70] Wong F.L., Francisco L., Togawa K., Bosworth A., Gonzales M., Hanby C., Sabado M., Grant M., Forman S.J., Bhatia S. (2010). Long-term recovery after hematopoietic cell transplantation: Predictors of quality-of-life concerns. Blood.

[71] Chiodi S., Spinelli S., Ravera G., Petti A.R., Van Lint M.T., Lamparelli T., Gualandi F., Occhini D., Mordini N., Berisso G. (2000). Quality of life in 244 recipients of allogeneic bone marrow transplantation. Br. J. Haematol..

[72] Bieri S., Roosnek E., Helg C., Verholen F., Robert D., Chapuis B., Passweg J., Miralbell R., Chalandon Y. (2008). Quality of life and social integration after allogeneic hematopoietic SCT. Bone Marrow Transplant..

[73] Hamilton B.K., Rybicki L., Dabney J., McLellan L., Haddad H., Foster L., Abounader D., Kalaycio M., Sobecks R., Dean R. (2014). Quality of life and outcomes in patients 60 years of age after allogeneic hematopoietic cell transplantation. Bone Marrow Transplant..

[74] Mielcarek M., Martin P.J., Leisenring W., Flowers M.E., Maloney D.G., Sandmaier B.M., Maris M.B., Storb R. (2003). Graft-versus-host disease after nonmyeloablative versus conventional hematopoietic stem cell transplantation. Blood.

[75] Couriel D.R., Saliba R.M., Giralt S., Khouri I., Andersson B., de Lima M., Hosing C., Anderlini P., Donato M., Cleary K. (2004). Acute and chronic graft-versus-host disease after ablative and nonmyeloablative conditioning for allogeneic hematopoietic transplantation. Biol. Blood Marrow Transplant..

[76] Bashey A., Zhang X., Sizemore C.A., Manion K., Brown S., Holland H.K., Morris L.E., Solomon S.R. (2013). T-cell-replete HLA-haploidentical hematopoietic transplantation for hematologic malignancies using post-transplantation cyclophosphamide results in outcomes equivalent to those of contemporaneous HLA-matched related and unrelated donor transplantation. J. Clin. Oncol..

[77] Burroughs L.M., O’Donnell P.V., Sandmaier B.M., Storer B.E., Luznik L., Symons H.J., Jones R.J., Ambinder R.F., Maris M.B., Blume K.G. (2008). Comparison of outcomes of HLA-matched related, unrelated, or HLA-haploidentical related hematopoietic cell transplantation following nonmyeloablative conditioning for relapsed or refractory Hodgkin lymphoma. Biol. Blood Marrow Transplant..

[78] Raiola A.M., Dominietto A., di Grazia C., Lamparelli T., Gualandi F., Ibatici A., Bregante S., Van Lint M.T., Varaldo R., Ghiso A. (2014). Unmanipulated haploidentical transplants compared with other alternative donors and matched sibling grafts. Biol. Blood Marrow Transplant..

[79] Gutman J.A., Ross K., Smith C., Myint H., Lee C.K., Salit R., Milano F., Delaney C., Gao D., Pollyea D.A. (2016). Chronic graft versus host disease burden and late transplant complications are lower following adult double cord blood versus matched unrelated donor peripheral blood transplantation. Bone Marrow Transplant..

[80] Konuma T., Tsukada N., Kanda J., Uchida N., Ohno Y., Miyakoshi S., Kanamori H., Hidaka M., Sakura T., Onizuka M. (2016). Comparison of transplant outcomes from matched sibling bone marrow or peripheral blood stem cell and unrelated cord blood in patients 50 years or older. Am. J. Hematol..

[81] Bensinger W.I., Martin P.J., Storer B., Clift R., Forman S.J., Negrin R., Kashyap A., Flowers M.E., Lilleby K., Chauncey T.R. (2001). Transplantation of bone marrow as compared with peripheral-blood cells from hla-identical relatives in patients with hematologic cancers. N. Engl. J. Med..

[82] Anasetti C., Logan B.R., Lee S.J., Waller E.K., Weisdorf D.J., Wingard J.R., Cutler C.S., Westervelt P., Woolfrey A., Couban S. (2012). Peripheral-blood stem cells versus bone marrow from unrelated donors. N. Engl. J. Med..

[83] Khoury H.J., Langston A.A., Kota V.K., Wilkinson J.A., Pusic I., Jillella A., Bauer S., Kim A.S., Roberts D., Al-Kadhimi Z. (2018). Ruxolitinib: A steroid sparing agent in chronic graft-versus-host disease. Bone Marrow Transplant..

[84] Jagasia M., Zeiser R., Arbushites M., Delaite P., Gadbaw B., Bubnoff N.V. (2018). Ruxolitinib for the treatment of patients with steroid-refractory GVHD: An introduction to the reach trials. Immunotherapy.

[85] Miklos D., Cutler C.S., Arora M., Waller E.K., Jagasia M., Pusic I., Flowers M.E., Logan A.C., Nakamura R., Blazar B.R. (2017). Ibrutinib for chronic graft-versus-host disease after failure of prior therapy. Blood.

[86] Schroeder T., Rachlis E., Bug G., Stelljes M., Klein S., Steckel N.K., Wolf D., Ringhoffer M., Czibere A., Nachtkamp K. (2015). Treatment of acute myeloid leukemia or myelodysplastic syndrome relapse after allogeneic stem cell transplantation with azacitidine and donor lymphocyte infusions—A retrospective multicenter analysis from the German cooperative transplant study group. Biol. Blood Marrow Transplant..

[87] Maury S., Lemoine F.M., Hicheri Y., Rosenzwajg M., Badoual C., Cherai M., Beaumont J.L., Azar N., Dhedin N., Sirvent A. (2010). CD4^+^CD25^+^ regulatory t cell depletion improves the graft-versus-tumor effect of donor lymphocytes after allogeneic hematopoietic stem cell transplantation. Sci. Transl. Med..

[88] Bejanyan N., Weisdorf D.J., Logan B.R., Wang H.L., Devine S.M., de Lima M., Bunjes D.W., Zhang M.J. (2015). Survival of patients with acute myeloid leukemia relapsing after allogeneic hematopoietic cell transplantation: A center for international blood and marrow transplant research study. Biol. Blood Marrow Transplant..

[89] Shah M.V., Jorgensen J.L., Saliba R.M., Wang S.A., Alousi A.M., Andersson B.S., Bashir Q., Ciurea S.O., Kebriaei P., Marin D. (2018). Early post-transplant minimal residual disease assessment improves risk stratification in acute myeloid leukemia. Biol. Blood Marrow Transplant..

[90] Platzbecker U., Middeke J.M., Sockel K., Mütherig A., Herbst R., Hänel M., Wolf D., Baldus C.D., Fransecky L., Noppeney R. (2017). Minimal-residual disease guided treatment with azacitidine in MDS/AML patients at imminent risk of relapse: Results of the prospective relaza2 trial. Blood.

[91] Kekre N., Kim H.T., Thanarajasingam G., Armand P., Antin J.H., Cutler C., Nikiforow S., Ho V.T., Koreth J., Alyea E.P. (2015). Efficacy of immune suppression tapering in treating relapse after reduced intensity allogeneic stem cell transplantation. Haematologica.

[92] Bar M., Sandmaier B.M., Inamoto Y., Bruno B., Hari P., Chauncey T., Martin P.J., Storb R., Maloney D.G., Storer B. (2013). Donor lymphocyte infusion for relapsed hematological malignancies after allogeneic hematopoietic cell transplantation: Prognostic relevance of the initial CD3+ T cell dose. Biol. Blood Marrow Transplant..

[93] De Lima M., Giralt S., Thall P.F., de Padua Silva L., Jones R.B., Komanduri K., Braun T.M., Nguyen H.Q., Champlin R., Garcia-Manero G. (2010). Maintenance therapy with low-dose azacitidine after allogeneic hematopoietic stem cell transplantation for recurrent acute myelogenous leukemia or myelodysplastic syndrome: A dose and schedule finding study. Cancer.

[94] Platzbecker U., Wermke M., Radke J., Oelschlaegel U., Seltmann F., Kiani A., Klut I.M., Knoth H., Rollig C., Schetelig J. (2012). Azacitidine for treatment of imminent relapse in MDS or AML patients after allogeneic HSCT: Results of the RELAZA trial. Leukemia.

[95] Craddock C., Labopin M., Robin M., Finke J., Chevallier P., Yakoub-Agha I., Bourhis J.H., Sengelov H., Blaise D., Luft T. (2016). Clinical activity of azacitidine in patients who relapse after allogeneic stem cell transplantation for acute myeloid leukemia. Haematologica.

[96] Pusic I., Choi J., Fiala M.A., Gao F., Holt M., Cashen A.F., Vij R., Abboud C.N., Stockerl-Goldstein K.E., Jacoby M.A. (2015). Maintenance therapy with decitabine after allogeneic stem cell transplantation for acute myelogenous leukemia and myelodysplastic syndrome. Biol. Blood Marrow Transplant..

[97] Schroeder T., Czibere A., Platzbecker U., Bug G., Uharek L., Luft T., Giagounidis A., Zohren F., Bruns I., Wolschke C. (2013). Azacitidine and donor lymphocyte infusions as first salvage therapy for relapse of AML or MDS after allogeneic stem cell transplantation. Leukemia.

[98] Schroeder T., Rautenberg C., Kruger W., Platzbecker U., Bug G., Steinmann J., Klein S., Hopfer O., Nachtkamp K., Kondakci M. (2018). Treatment of relapsed AML and MDS after allogeneic stem cell transplantation with decitabine and DLI-a retrospective multicenter analysis on behalf of the German cooperative transplant study group. Ann. Hematol..

[99] Chen Y.B., Li S., Lane A.A., Connolly C., Del Rio C., Valles B., Curtis M., Ballen K., Cutler C., Dey B.R. (2014). Phase I trial of maintenance sorafenib after allogeneic hematopoietic stem cell transplantation for FMS-like tyrosine kinase 3 internal tandem duplication acute myeloid leukemia. Biol. Blood Marrow Transplant..

[100] Schlenk R., Döhner K., Salih H., Kündgen A., Fiedler W., Salwender H.-J., Westermann J., Götze K.S., Horst H.-A., Wulf G. (2015). Midostaurin in combination with intensive induction and as single agent maintenance therapy after consolidation therapy with allogeneic hematopoietic stem cell transplantation or high-dose cytarabine. Blood.

[101] DiNardo C.D., Rausch C.R., Benton C., Kadia T., Jain N., Pemmaraju N., Daver N., Covert W., Marx K.R., Mace M. (2018). Clinical experience with the Bcl2-inhibitor venetoclax in combination therapy for relapsed and refractory acute myeloid leukemia and related myeloid malignancies. Am. J. Hematol..

[102] Stein E.M., DiNardo C.D., Pollyea D.A., Fathi A.T., Roboz G.J., Altman J.K., Stone R.M., DeAngelo D.J., Levine R.L., Flinn I.W. (2017). Enasidenib in mutant IDH2 relapsed or refractory acute myeloid leukemia. Blood.

[103] Davids M.S., Kim H.T., Bachireddy P., Costello C., Liguori R., Savell A., Lukez A.P., Avigan D., Chen Y.B., McSweeney P. (2016). Ipilimumab for patients with relapse after allogeneic transplantation. N. Engl. J. Med..

[104] Goodyear O.C., Dennis M., Jilani N.Y., Loke J., Siddique S., Ryan G., Nunnick J., Khanum R., Raghavan M., Cook M. (2012). Azacitidine augments expansion of regulatory T cells after allogeneic stem cell transplantation in patients with acute myeloid leukemia (AML). Blood.

[105] El-Jawahri A., Pidala J., Inamoto Y., Chai X., Khera N., Wood W.A., Cutler C., Arora M., Carpenter P.A., Palmer J. (2014). Impact of age on quality of life, functional status, and survival in patients with chronic graft-versus-host disease. Biol. Blood Marrow Transplant..

[106] Grunwald M.R., Zimmerman M.K.A., Boselli D., Bohannon L.M., Robinson M.M., Peters D.T., Ai J., Knight T.G., Trivedi J., Plesca D. (2017). Frontline azacitidine as a bridge to allogeneic transplantation in acute myeloid leukemia. Blood.

